# Predictive Roles of Three-Dimensional Psychological Pain, Psychache, and Depression in Suicidal Ideation among Chinese College Students

**DOI:** 10.3389/fpsyg.2017.01550

**Published:** 2017-09-12

**Authors:** Huanhuan Li, Rong Fu, Yingmin Zou, Yanyan Cui

**Affiliations:** Department of Psychology, Renmin University of China Beijing, China

**Keywords:** three-dimensional psychological pain, psychache, depression, suicidal ideation, college student

## Abstract

How to develop an effective screening instrument for predicting suicide risk is an important issue in suicidal research. The aim of the present research was to explore the predictive roles of three screening measures in the evaluation of preexisting suicide risk factors in a sample of undergraduate students. We assessed 1,061 students using the Beck depression and suicidal ideation scales (BDI-I) (BSI), the Psychache Scale (PAS), and the three-dimensional Psychological Pain Scale (TDPPS). Simultaneous multivariate regression analysis showed that the predictive values of pain avoidance scores and BDI scores for suicidal ideation were more significant than that of the PAS scores. Subsequently, 42 patients with major depressive disorder (MDD), 39 students with subthreshold depression (SD), and 18 healthy controls were voluntarily recruited. Students with SD were divided into high suicidal ideation (HSI-SD) and low suicidal ideation (LSI-SD) groups. Pain avoidance scores and BDI scores differed significantly among the MDD, HSI-SD, LSI-SD, and healthy control groups. Pain avoidance and BSI scores were significantly higher in the MDD and HSI-SD groups than those in the LSI-SD and healthy control groups. However, no significant difference was observed in BDI scores between the HSI-SD and LSI-SD groups. Pain avoidance and depression, rather than psychache, may be promising predictors of suicidal ideation in a Chinese young adult population.

## Introduction

Suicide is a substantial public health concern worldwide, and it poses a major challenge to health psychologists because of its limited predictability. In China, suicide has become the leading cause of death among young adults (those aged 15–34 years), and at a mean annual suicide rate of about 23 per 100,000 people, it is ~2 to 3 times greater than the global average (Phillips et al., 2002). The development of effective screening measures for suicidal behavior, particularly in young adults and at-risk clinical populations [e.g., patients with major depressive disorder (MDD)], is thus important.

## Backgrounds

Although, depression has been considered to be a major factor influencing suicidality, individuals with no depression can show suicidal behavior, and not all depressive patients think of ending their lives (Nock et al., 2010). The presence of depression alone is not sufficient for the prediction of suicidality (Pompili, 2010). Recently, the psychological pain model first proposed by Shneidman (1998) that suicide can occur when profound psychological pain becomes unbearable has received greater attention.

Psychological pain, or psychache, results from “the introspective experience of negative emotions such as dread, despair, fear, grief, shame, guilt, frustrated love, loneliness, and loss” (Shneidman, 1998). Psychological pain in comparison with depression has a more statistically significant predictive effect on suicidal ideation and suicide attempts among college students (Troister and Holden, 2010) and homeless people (Patterson and Holden, 2012). Suicidal ideation and suicide attempts are associated significantly with higher levels of psychological pain (Levi et al., 2008; van Heeringen et al., 2010). Psychological pain has been shown to mediate the effect of depression (Holden et al., 2001) on suicidality. Although the levels of psychological pain and depression were found to be significantly higher in individuals with histories of suicidal attempt than in those with no such history, psychological pain had a large effect size and the effect sizes of depression were moderate (Pereira et al., 2010). In a 2-year longitudinal study (Troister and Holden, 2012), baseline psychological pain levels and changes in these levels over time were unique statistical predictors of suicidal ideation, whereas depression was not. In a recent study (Troister et al., 2015), the psychological pain displayed superior performance in accurately identifying suicide risk than depression.

It is worthy of note that most individuals suffering from intense psychological pain do not commit suicide. For a suicidal person, psychological pain can become intolerable and unacceptable once a certain threshold is crossed, and the idea of death is perceived as a solution enabling escape from this unbearable pain (Shneidman, 1998, 2001, 2005). This seemingly contradictory phenomenon may be explained by suicide requiring both intolerable psychological pain and the idea of death as a solution (Shneidman, 2005). Thus, the internal construct of suicidality may involve increased motivation to avoid unbearable pain, which may be a dominant precursor of suicidal behavior (Li et al., 2014; Xie et al., 2014). This view was supported by a fMRI study, which showed that MDD patients with painful feeling-evoking showed a significant reduced frontal cortex activity compared to health controls. However, their frontal cortex activity showed a significant increase and recovery to normal state when MDD patients plan and act on suicidal impulses (Reisch et al., 2010). These findings suggested that suicidal behavior may be goal-directed, and the goal may be to escape from psychological pain.

Although various studies have underscored the importance of psychological pain, painful feelings were mainly assessed using any of the instruments available for the measurement of psychological pain. For example, the most widely adopted scale for the assessment of psychological pain is the Psychache Scale (PAS), which briefly assesses chronic, free-floating, non-situation specific, psychological pain caused by the frustration of vital psychological needs (Holden et al., 2001). Despite its high reliability and validity, as demonstrated in a large number of studies (Mills et al., 2005; Flamenbaum and Holden, 2007; Troister and Holden, 2010, 2012; Patterson and Holden, 2012), it primarily addresses subjective feelings of psychological pain, with few items that assess pain intensity. Notably, the Mee-Bunney psychological pain assessment Scale (Mee et al., 2011) includes one item that encompasses death as the only way to stop pain, but the contribution of this single item has not been reported.

Considering that psychological pain can reasonably be proposed to represent a multidimensional construct, including cognitive appraisal, bodily symptoms, expression, feelings, and action tendencies (Orbach et al., 2003; Scherer, 2005), we proposed three-dimensional psychological pain model. First, the cognitive appraisal of psychological pain, defined as pain arousal, represents pain derived from the memory of past traumatic experiences such as bereavement, failure/frustration, or social exclusion. Second, the subjective and bodily symptoms related to psychological pain is defined as painful feelings that most existing measures focused on. Third, considering that suicidal motivation, including internal perturbation-based reasons, manipulative motivations, extrapunitive motivations, and self-destructive motivations were closely associated with the tendency to suicidal acts (Holden et al., 2001), active pain avoidance may be especially important with regard to suicide, as it represents a strong motivation or action tendency to commit suicide as the only means of pain relief. Our previous work found that only pain avoidance could predict suicidal acts among MDD patients, while depression and psychache measured by the PAS could not (Li et al., 2014). However, very few studies have explored the role of pain avoidance motivation in predicting suicidal ideation or action among adolescents and young adults.

In summary, with the goal of exploring the predictive effects for identifying suicide risk among depression, psychache, and pain avoidance, we evaluate the TDPPS which consists of three subscales, pain arousal, painful feelings, and active pain avoidance (Li et al., 2014), BDI-I, and PAS for their respective abilities to individually screen for undergraduate students with a high risk of suicide. We also compared the differences in the BDI-I, PAS, and TDPPS among patients with MDD, college students with subthreshold depression (SD), and healthy control students. Given that a SD individual has clinically relevant depressive symptoms, such as scoring above a cut-off score on a self-rating depression scale, and he or she is without meeting criteria for a full-blown MDD (Cuijpers and Smit, 2004), all SD students in the present study scored above the cut-off point “17” of the BDI which is recommended as an index of clinically significant depression (Ye et al., 2013).

We hypothesized that the predictive power of pain avoidance measured by the TDPPS on suicidal ideation was greater than psychache measured by the PAS and depression measured by the BDI-I in this population, and that pain avoidance can discriminate between MDD or SD individuals with high suicidal ideation (HSI-SD) and SD with low suicidal ideation (LSI-SD) or healthy individuals, whereas depression and psychache cannot.

## Study 1: exploring the relationships among psychological pain, psychache, depression, and suicidal ideation

Study 1 was conducted to further investigate the predictive roles of pain avoidance (measured by the TDPPS), psychache (measured by the PAS), and depression (measured by the BDI-I) in suicidal ideation among Chinese college students. The study sought to determine whether the presence of pain avoidance was correlated significantly with suicidal ideation, and whether the predictive power of pain avoidance was greater than those of PAS and BDI-I scores, in this population.

### Methods

#### Participants

A total of 1,185 undergraduate students from four universities in China participated voluntarily in this study with no compensation. The Institutional Review Board of Renmin University of China approved the study protocol. Inclusion criteria were as follows: (a) majors in social science (philosophy, economics, business administration, education, law, and politics) and natural science (math, physics, chemistry, biology, life sciences, and medicine); (b) educational level ranging from grade 13 to grade 16; (c) age 17–23 years; and (d) normal hearing and vision. Exclusion criteria was a self-report previous history of mental diseases (schizophrenia, mood disorders, anxiety disorders, etc.) or chronic physical diseases (cardiovascular disease, tumor, etc.) diagnosed by experienced clinicians.

All participants gave informed consent prior to the beginning of the study. The participants were told that their responses were anonymous and they could withdraw at any time.

Enrollment was conducted during two time periods: All participants in sample 1 were recruited in April–July 2013.

#### Measures

##### Depression

The Beck depression inventory I (BDI-I; Beck, 1988) is a 21-item self-report scale and is used to evaluate the severity of depression, typified by phrases such as “I do feel guilty.” Responses to each item are rated on a 4-point scale ranging from 0 (no symptom) to 3 (most severe) and are based on feelings and experiences from the past week. The Chinese version of the BDI-I which has demonstrated high reliability and validity in a number of studies conducted in China (Zhang et al., 1990) was employed in the present study. We considered BDI-I cut-off scores <4 to be indicative of non-depression and scores ≥17 to be indicative of clinically significant depression (Ye et al., 2013). In the current sample, the internal consistency of the BDI-I was excellent (Cronbach's α = 0.93).

##### Suicidal ideation

The 19-item BSI (Beck and Steer, 1991) is used to evaluate the severity of current wishes and plans to commit suicide, typified by phrases such as “To what extent you want to live?.” Participants rate descriptions of each item on a 3-point scale that ranges from 0 (not at all) to 2 (extremely) according to their experiences in the past week (current, BSI-C) and during the most severe period of suicidal ideation (worst, BSI-W). The Chinese version of the BSI which was translated and revised by the Beijing Suicide Research and Prevention Center (Li et al., 2010) was used in the present study. The internal consistency coefficients for the 2 subscales were excellent in the current sample (BSI-C, Cronbach's α = 0.96; BSI-W, Cronbach's α = 0.94).

##### Psychache

The 13-item self-report PAS (Holden et al., 2001) was used to measure subjective feelings of psychological pain, as defined by Shneidman (1998), using 5-point Likert ratings (1 = not at all, 5 = extremely). Example items are “I seem to ache inside” and “I'm hurt because I feel empty.” In previous studies, the PAS has shown excellent reliability (Cronbach's α > 0.90) (Flamenbaum and Holden, 2007; Troister and Holden, 2010; Patterson and Holden, 2012). Holden et al. (2001) also reported that this instrument enables successful differentiation between suicide attempters and non-attempters. With the authors' permission, we developed a Chinese version of the PAS using translation and back-translation, similar to the procedure used to develop the TDPPS. In the current sample, the internal consistency of this instrument was excellent (Cronbach's α = 0.96).

##### Three-dimensional psychological pain

The TDPPS, a 17-item measure representing the three-dimensional model of psychological pain, was also administered. Its three dimensions are pain arousal (eight items), painful feelings (six items), and active pain avoidance (three items), typified by phrases such as “Whenever I think of my serious shortcomings, I feel a great deal of pain,” “My experience was very unfortunate, which makes me feel a great deal of pain,” “The pain feels like my heart crunched up,” “For me, suicide is relief, because my pain, the psychological pain, would stop,” and “My pain hurts so badly that death could be the only way to escape from it.” The three subscale scores were composite scores made up of current and worst pain in the past time. Responses to each item are rated on a 5-point Likert scale [describes me “not at all” (1) to “very well” (5)].

In the current sample, the Cronbach's α coefficient for the total TDPPS was high (0.93). The internal consistency of the three subscales was also excellent (pain arousal, Cronbach's α = 0.85; painful feelings, Cronbach's α = 0.86; pain avoidance, Cronbach's α = 0.77). EFA yielded three components, and all three factors had characteristic root values >1.0, which together explained 55.5% of the total variance. For the confirmatory factor analysis, the model fitting indices met statistical standards adequately: χ^2^_(116)_ = 454.93, *p* < 0.001; NFI = 0.90; NNFI = 0.96; CFI = 0.97; GFI = 0.90; SRMR = 0.056; RMSEA = 0.079 (90% CI, 0.072–0.086).

#### Procedure

All participants were asked to complete the set of self-report instruments in a specified order, BDI-I, BSI, PAS, and TDPPS, followed by demographic questions. This order was selected to minimize the psychological difficulty of answering questions by exposing participants to the least intrusive questions first (Patterson and Holden, 2012).

#### Data analysis

The statistical analysis was performed using the Statistical Package for Social Sciences (SPSS, version 17.0 for Windows; Chicago, IL). Descriptive analyses were performed on all variables. Using data from all 1,061 participants, bivariate associations between suicidal ideation and demographic variables, depression, psychache, total TDPPS scores, pain avoidance, painful feelings, and pain arousal subscale scores were assessed using regression analyses. Variables with *p* < 0.05 were included in a simultaneous multivariate regression model, with suicidal ideation as the dependent variable, to evaluate the significance of each predictor after controlling for all the other predictors.

### Results

#### Demographic characteristics

The original sample consisted of 1,185 Chinese college students [554 (47%) men, 602 (51%) women, 29 (2%) undisclosed gender] with a mean (*M*) age of 21.03 [standard deviation (*SD*) = 2.46] years. Of those, 124 were excluded due to missing data or specific reaction tendencies (the same response for different items), resulting in a final sample of 1,061 students. Subject breakdown by university type was as follows: 506 with average age 20.66 (*SD*, 2.28) and 230 with an average age of 21.89 (*SD*, 2.91) were from two comprehensive, research-orientated universities, 203 with an average age of 22.53 (*SD*, 2.56) were from a research-oriented university of engineering science and technology, and 246 with an average age of 19.75 (*SD*, 0.89) were from a medical university.

#### Relationships among psychological pain, depression, and suicidal ideation

The results are summarized in Tables 1–3. All of the three instruments used showed similar significant correlations, with TDPPS, especially pain avoidance showing slightly higher significant correlations with suicidal ideation at one's worst point and current suicidal ideation.

**Table 1:** Criterion-predictor correlations.

	**BDI-I-CV**	**PAS**	**TDPPS**	**Painful feelings**	**Pain arousal**	**Pain avoidance**	**BSI-C**	**BSI-W**
BDI-I-CV	–							
PAS	0.67^**^	–						
TDPPS	0.57^**^	0.73^**^	–					
Painful feelings	0.46^**^	0.64^**^	0.92^**^	–				
Pain arousal	0.57^**^	0.69^**^	0.94^**^	0.74^**^	–			
Pain avoidance	0.41^**^	0.49^**^	0.55^**^	0.36^**^	0.48^**^	–		
BSI-C	0.39^**^	0.38^**^	0.33^**^	0.19^**^	0.31^**^	0.54^**^	–	
BSI-W	0.42^**^	0.44^**^	0.47^**^	0.36^**^	0.41^**^	0.63^**^	0.64^**^	–

*N = 1,061*.

** *p < 0.01*.

*BDI-I-CV, The Beck Depression Inventory-I Chinese Version; PAS, the Psychache Scale; TDPPS, the Three-Dimensional Psychological Pain Scale; BSI-C, the Beck Scale for current suicide ideation; BSI-W, the Beck Scale for suicide ideation at one's worst Point*.

**Table 2:** Summary of simultaneous multivariate regression results for variables predicting BSI-W scores.

**Variable**	**B**	**SEB**	**β**
Gender	1.98	0.76	0.08^**^
Educational level	−0.38	0.19	−0.07^*^
Degree subject	0.65	0.24	0.09^**^
Depression	0.38	0.06	0.21^***^
Psychache	0.02	0.04	0.02
Pain arousal	−0.17	0.12	−0.09
Painful feelings	0.23	0.11	0.11^*^
Pain avoidance	0.88	0.22	0.18^***^

* p < 0.05;

** p < 0.01;

*** *p < 0.001*.

**Table 3:** Summary of simultaneous multivariate regression results for variables predicting BSI-C scores.

**Variable**	**B**	**SEB**	**β**
Age	−0.11	0.05	−0.07^*^
Educational level	−0.19	0.24	−0.03
Depression	0.27	0.08	0.12^**^
Psychache	0.01	0.05	0.01
Pain arousal	0.05	0.15	0.02
Painful feelings	−0.10	0.15	−0.04
Pain avoidance	0.74	0.29	0.12^**^

* p < 0.05;

** *p = 0.01*.

Bivariate associations between BSI scores and demographic variables (i.e., age, gender, and academic major) and between BSI scores and clinical variables (i.e., the PAS scores, the BDI scores, the TDPPS total scores, painful feelings, pain arousal, and pain avoidance subscale scores) were assessed using regression analyses. With the exception of age, the other predictive variables were associated significantly with BSI-W scores. With the exception of academic major and gender, the other predictive variables were associated significantly with BSI-C scores.

To determine the relative importance of each predictor after controlling for the other predictors, variables significantly correlated with BSI scores were included in a simultaneous multivariate regression model with BSI scores as the dependent variable. Five variables—academic major (β = 0.09, *p* < 0.01), gender (β = 0.08, *p* < 0.01), depression (β = 0.21, *p* < 0.001), pain avoidance (β = 0.18, *p* < 0.001), and painful feelings (β = 0.11, *p* < 0.05)—were significant predictors of BSI-W score, with the two most important predictors being pain avoidance and depression (Table 2). Three variables—depression (β = 0.12, *p* = 0.01), pain avoidance (β = 0.12, *p* = 0.01), and age (β = −0.07, *p* < 0.05)—were significant predictors of BSI-C scores, with the 2 most important predictors also being pain avoidance and depression.

## Study 2: comparison of the beck suicide ideation scale, beck depression inventory I and the three-dimensional psychological pain scale (TDPPS) among MDD patients, SD students and health controls

The research question addressed in study 2 was whether the predictive effect of pain avoidance for suicidality would be significantly greater than that of depression.

### Methods

#### Participants

In study 2, the sample consisted of 42 patients with MDD, 39 SD students (BDI-I scores >17), and 18 healthy students (BDI-I scores <4).

Fifty-seven undergraduate students were recruited from four universities by advertisement and were compensated monetarily. They completed the BSI, BDI-I, PAS, and TDPPS. Among them, 39 students rating a BDI-I score of 17 or above were assessed by an experienced psychiatrist with a clinical interview. When they were without meeting DSM-IV criteria for a full-blown MDD, they were considered as SD students. According to Beck and Steer (1991) guidelines, respondents with BSI-C scores ≥2 and/or BSI-W scores >16 can be regarded as having high suicidal ideation (HSI). Further, current suicide ideators are likely to have been ideators in the past (94%), whereas non-ideators at their worst points are not likely to be current ideators (1%) (Beck et al., 1997). Thus, to establish a clear difference, participants with high BSI-C *and/or* BSI-W scores were assigned to the HSI group and those with low scores on both BSI subscales (BSI-C scores <2 and BSI-W scores ≤10) were assigned to the LSI group. On the basis of BSI scores, SD students were allocated to the HSI-SD (*n* = 18) and LSI-SD (*n* = 17) groups.

Forty-two patients with MDD were recruited from the Sixth Hospital of Peking University (Table 4). Assessments were conducted to determine their eligibility. Inclusion criteria were as follows: (a) diagnosis of MDD was confirmed by an experienced psychiatrist based on the criteria of the Diagnostic and Statistical Manual of Mental Disorders, 4th Edition, with no history of hypomania or mania; (b) educational level exceeding junior middle school; (c) age 18–55 years; and (d) normal hearing and vision. Exclusion criteria were: (a) Mini-Mental State Examination (MMSE; Crum et al., 1993) score <32, (b) combined use of antipsychotic drugs, (c) brain injury or brain organic disease, (d) severe mental illness (i.e., schizophrenia, bipolar disorder), (e) mental retardation, and (f) severe and unstable somatic disease. The Institutional Review Board of the Sixth Hospital of Peking University approved the study protocol, and all participants provided informed consent.

**Table 4:** Demographic and clinical characteristics of MDD patients.

**Characteristic**	**MDD (*n* = 42)**
**AGE**	28.43 (8.79)
**GENDER**	
Male	18 (42.9%)
Female	24 (57.1%)
Educational level (years)	15.7 (2.6)
**MARITAL STATUS**	
Married	14 (33.3%)
Single	27 (64.3%)
Divorced	1 (2.4%)
Age of first onset of depression (years)	26.5 (8.53)
**EPISODES OF DEPRESSION**	
First episode	17 (40.5%)
Recurrent	25 (59.5%)
**CURRENT MEDICATION USE**	
SSRI	15 (35.7%)
SNRI	3 (7.1%)
SSRI & SNRI	9 (21.5%)
None	15 (35.7)
**SELF-REPORTED SUICIDE RISK**	
Suicidal ideation	19 (100%)
Suicidal intent	9 (47.4%)
Suicide attempt	1 (5.3%)

The MDD and SD groups were recruited from September 2014–December 2015.

#### Data analysis

Questionnaire data among MDD, SD, and healthy control groups was compared using MANOVA. Partial eta-squared (η^2^) values were calculated as measures of effect size.

### Results

#### Demographic characteristics

Demographic characteristics and questionnaire scores of the MDD patient sample in Table 1. Gender did not differ significantly among the MDD, HSI-SD, LSI-SD, and the healthy control groups, but age did differ [*F*_(3, 95)_ = 8.05, *p* < 0.001]. As suicide rates generally increase with age (Shah et al., 2009), MANOVA with age as a covariate was done to all the outcome measures.

#### Intergroup differences in questionnaire scores

Significant differences among the MDD, HSI-SD, LSI-SD, and health control groups were observed in suicidal ideation at its worst point [*F*_(3, 95)_ = 17.73, *p* < 0.001], current suicidal ideation [*F*_(3, 95)_ = 47.05, *p* < 0.001], the BDI-I scores, the total scores of TDPPS, pain arousal, painful feelings, and pain avoidance subscale scores [*F*_(3, 95)_ = 23.67, *p* < 0.001].

*Post-hoc* analysis showed that painful feelings, pain arousal, and BDI-I scores were significantly higher in the MDD and SD groups than those in the healthy controls, but did not differ significantly between the MDD, HSI-SD, and LSI-SD groups (*p* > 0.05). Notably, pain avoidance subscale scores were significantly higher in the HSI-SD group (HSI-SD vs. LSI-SD: *t* = 3.38, *p* < 0.001; HSI-SD vs. healthy controls: *t* = 4.44, *p* < 0.001) and the MDD group (MDD vs. LSI-SD: *t* = 4.61, *p* < 0.001; MDD vs. healthy controls: *t* = 5.68, *p* < 0.001), and that pain avoidance subscale scores did not differ significantly between the LSI-SD group and healthy controls (*t* = 1.07, *p* > 0.05).

According to Cohen's (1992) guideline, the BDI-I scores, the total scores of TDPPS and pain avoidance subscale scores which marginally differed significantly among the 4 groups had medium effects (partial η^2^ = 0.45–0.62) (Table 5).

**Table 5:** Differences in questionnaire scores among the MDD, HSI, LSI, and healthy control groups.

	**MDD**	**HSI**	**LSI**	**Healthy control**	***F*/χ^2^**	***P***	**Partial η^2^**
	***n* = 42**	***n* = 18**	***n* = 21**	***n* = 18**			
BDI	30.12 (11.41)^***^^###^^$^	24.94 (5.71)^***^	21.71 (5.13)^***^	1 (1.28)	2.50	0.09	0.07
BSI-C	9.90 (7.94)^***^^###^	7.50 (3.92)^***^^###^	0.67 (0.89)	0 (0)	8.94	<0.001	0.21
BSI-W	19.73 (8.68)^***^^###^	16.82 (6.32)^***^^###^	9.31 (3.77)^*^	0 (0)	8.61	<0.001	0.21
Total TDPPS	60.14 (10.50)^***^^###^	58.39 (8.05)^***^^##^	50.48 (6.91)^***^	30.11 (8.64)	2.29	0.11	0.07
Pain arousal	27.62 (5.33)^***^^#^	27.11 (4.80)^***^	24.43 (3.75)^***^	13.22 (4.33)	1.01	0.37	0.03
Painful feelings	23.79 (3.77)^***^^#^	23.78 (2.98)^***^	21.67 (3.47) ^***^	13.72 (5.15)	0.37	0.70	0.01
Pain avoidance	8.74 (3.34)^***^^###^	7.50 (2.41)^***^^###^	4.38 (1.83)	3.06 0.24	6.46	<0.01	0.16

* *p < 0.05*,

^**^p < 0.01,

*** *p < 0.001 vs. healthy controls*.

# *p < 0.05*,

## *p < 0.01*,

### *p < 0.001 vs. LSI group*.

$ *p < 0.05 vs. HSI group*.

*HSI, high suicidal ideation; LSI, low suicidal ideation; BDI, Beck Depression Inventory; BSI-C, Beck Suicidal Ideation-Current Scale; BSI-W, Beck Suicidal Ideation-Worst scale; TDPPS, 3-Dimensional Psychological Pain Scale*.

## General discussion

Psychological pain has been identified as a high risk factor for suicide with greater predictive power than depression (Olié et al., 2010; Pereira et al., 2010; Troister and Holden, 2010; Li et al., 2014; Troister et al., 2015). Using data from a large sample of college students, we demonstrated that pain avoidance showed very different tendencies between students at high and low risks of suicide. MDD patients and HSI-SD students had significantly higher pain avoidance subscale scores than LSI-SD students. However, BDI scores did not differ significantly between these two high suicidal risk groups (MDD, HSI-SD) and the LSI-SD group.

In study 1, our results indicated that depression and pain avoidance were the 2 most significant predictors of current suicidal ideation and suicidal ideation at its worst point for an undergraduate student, whereas psychache was not a statistical predictor. When controlling for depression and pain avoidance subscale scores, psychache, and painful feelings subscale scores were not significantly associated with suicidal ideation in the multivariate regression analysis. These findings are partially consistent with our previous work showing that for MDD patients, pain avoidance is a primary predictor of worst suicidal ideation and suicide attempts, but depression and psychache were not (Li et al., 2014). This discrepancy on the predictive roles of pain avoidance and depression on worst suicidal ideation between undergraduate students and MDD patients suggested that even those students without meeting criteria for MDD, with strong motivation to use suicide as a means of escaping painful situations or with high levels of depression, are at high risk of suicide. However, for MDD patients who already scored a very high level of depression, pain avoidance may be a stronger predictor to identify those at high risk for suicidal attempt. Based on that worst suicidal ideation was a better predictor of suicide attempts in hospitalized patients than current suicidal ideation (Beck et al., 1999), it can be deduced that three-dimensional psychological pain may also be a promising predictor of suicide acts in undergraduate student.

Further, depression was also an important predictor of suicidal ideation rather than psychache in study 1, suggesting that the inability to experience pleasure from activities usually found enjoyable (Abramson et al., 1989) leads to suicidal ideation. Depression is said to be commonly present in people who commit suicide. The predictive role of depression on suicidality was also supported by a cross-cultural study showing that depression distinguished attempters from controls both in Chinese and US cultures (Stewarta et al., 2006). However, our results is not consistent with a recent study showing that depression and psychache have significant diagnostic accuracy for suicidal ideation and suicide attempt; in addition, psychache was the psychological variable associated most strongly with suicidality in a large sample of undergraduate students (Troister et al., 2015). Considering that the same measure of psychache (PAS) was used in Troister's study (2015) and the present study, cultural background may contribute to the discrepancy in the effect of psychache on suicidality. On the one hand, Chinese individuals living in an authoritarian culture must develop defenses and emotional attitudes to cope adaptively and to achieve surface harmony (Jie, 2011); thus, they may tend to repress, rather than express subjective painful feelings, which results in a lower painful feeling subscale scores than their real feelings. In the US context, people do not avoid expressing pain and have tendency to choose negative coping, such as withdraw if pain becomes intense (Calvillo and Flaskerud, 1991). Thus, Western people may score high painful feeling scores close to their real feelings. Self-reported painful feelings scores in Western people may predict suicide much stronger than those in Chinese people when intense psychological pain became unbearable. On the other hand, suicide may have a different meaning in the Chinese context from the US context; it may be taken as an act that helps someone to achieve what he/she could not during his/her life time (Zhang et al., 2005), such as relief (*jietuo*) or reincarnation (*tou tai*). In the US context, religion, such as Christianity can potentially serve as a protective factor against the suicidal act (Gearing and Lizardi, 2009). Thus, Chinese people are more able to accept suicide as a means of escaping from unbearable painful feelings.

In study 2, although the BDI-I, painful feelings, and pain arousal scores could discriminate between depressed individuals (i.e., MDD and SD) and healthy controls, they did not differ significantly between the HSI-SD and LSI-SD groups. Only pain avoidance scores differed significantly between the two high suicide risk groups (i.e., MDD and HSI-SD) and the LSI-SD group, suggesting pain avoidance was more sensitive than depression in detecting suicide risk among young adults with SD. These findings imply that the TDPPS, especially the pain avoidance subscale, is a diagnostically efficient measure for the identification of undergraduate students at the greatest risk of suicide.

The present research has several limitations. First, the participants of sample 1 were college students, who may not be representative of the general population. Second, the samples of MDD (*n* = 42) and SD (*n* = 35) participants were small. As a consequence, the conclusions may not be generalized to SD or MDD populations. Third, this research was cross-sectional, and longitudinal studies should be conducted to investigate whether the TDPPS can predict the effects of interventions. Finally, hopelessness, a well-defined predictor of suicidality, was not assessed in our studies. Future research that includes consideration of hopelessness is needed to more fully understand the superior predictive effects of psychological pain on suicidal ideation and suicidal act among college students.

## Ethics statement

This study was carried out in accordance with the recommendations of Ethic of guidelines, The Institutional Review Board of Renmin University of China with written informed consent from all participants. The Institutional Review Board of Renmin University of China and The Institutional Review Board of the Sixth Hospital of Peking University approved the study protocol for this study. All participants gave written informed consent in accordance with the Declaration of Helsinki.

## Author contributions

HL: Study design, data analysis and manuscript prepared. RF: sample collecting. YZ: sample collecting and data analysis. YC: sample collecting.

### Conflict of interest statement

The authors declare that the research was conducted in the absence of any commercial or financial relationships that could be construed as a potential conflict of interest.

## References

[B1] Abramson L. Y. Metalsky G. I. Alloy L. B. (1989). Hopelessness depression: a theory-based subtype of depression. Psychol. Rev. 96, 358–372. 10.1037/0033-295X.96.2.358

[B2] Beck A. T. (1988). Psychometric properties of the Beck Depression Inventory: twenty-five years of evaluation. Clin. Psychol. Rev. 8, 77–100. 10.1016/0272-7358(88)90050-5

[B3] Beck A. T. Brown G. K. Steer R. A. (1997). Psychometric characteristics of the Scale for suicide ideation with psychiatric outpatients. Behav. Res. Ther. 35, 1039–1046. 10.1016/S0005-7967(97)00073-9 9431735

[B4] Beck A. T. Brown G. K. Steer R. A. Dahlsgaard K. K. Grisham J. R. (1999). Suicide ideation at its worst point: a predictor of eventual suicide in psychiatric outpatients. Suicide Life Threat. Behav. 29, 1–9. 10322616

[B5] Beck A. T. Steer R. A. (1991). Mannual for Beck Scale for Suicide Ideation. San Antonio, TX: Psychological Corporation.

[B6] Calvillo E. R. Flaskerud J. H. (1991). Review of literature on culture and pain of adults with focus on mexican-americans. J. Transcult. Nurs. 2, 16–23. 10.1177/104365969100200203 2043291

[B7] Cohen J. (1992). A power primer. Psychol. Bull. 112, 155–159. 10.1037/0033-2909.112.1.155 19565683

[B8] Crum R. M. Anthony J. C. Bassett S. S. Folstein M. F. (1993). Population-based norms for the Mini-Mental State Examination by age and educational level. JAMA 269, 2386–2391. 10.1001/jama.1993.03500180078038 8479064

[B9] Cuijpers P. Smit F. (2004). Subthreshold depression as a risk indicator for major depressive disorder: a systematic review of prospective studies. Acta Psychiatr. Scand. 109, 325–331. 10.1111/j.1600-0447.2004.00301.x 15049768

[B10] Flamenbaum R. Holden R. R. (2007). Psychache as a mediator in the relationship between perfectionism and suicidality. J. Couns. Psychol. 54, 51–61. 10.1037/0022-0167.54.1.51

[B11] Gearing R. E. Lizardi D. (2009). Religion and suicide. J. Relig. Health 48, 332–341. 10.1007/s10943-008-9181-2 19639421

[B12] Holden R. R. Mehta K. Cunningham E. J. McLeod L. D. (2001). Development and preliminary validation of a scale of psychache. Can. J. Behav. Sci. 33, 224–232. 10.1037/h0087144

[B13] Jie Z. (2011). Working with chinese patients: are there conflicts between Chinese culture and psychoanalysis?. Int. J. Appl. Psychoanal. Stud. 8, 218–226. 10.1002/aps.304

[B14] Levi Y. Horesh N. Fischel T. Treves I. Or E. Apter A. (2008). Mental pain and its communication in medically serious suicide attempts: an “impossible situation.” J. Affect. Disord. 111, 244–250. 10.1016/j.jad.2008.02.022 18436309

[B15] Li H. Xie W. Z. Luo X. W. Rong F. Ying X. Y. Wang X. (2014). Clarifying the role of psychological pain in the risks of suicidal ideation and suicidal acts among patients with major depressive episodes. Suicide Life Threat. Behav. 44, 78–88. 10.1111/sltb.12056 24106764

[B16] Li X.-Y. Phillips M. R. Tong Y.-S. Li K.-J. Zhang Y.-L. Zhang Y.-P. (2010). Reliability and validity of the Chinese version of beck suicide ideation scale (BSI-CV) in adult community residents. Chin. Ment. Health J. 24, 250–255.

[B17] Mee S. Bunney B. G. Bunney W. E. Hetrick W. Potkin S. G. Reist C. (2011). Assessment of psychological pain in major depressive episodes. J. Psychiatr. Res. 45, 1504–1510. 10.1016/j.jpsychires.2011.06.011 21831397

[B18] Mills J. F. Green K. Reddon J. R. (2005). An evaluation of the psychache scale on an offender population. Suicide Life Threat. Behav. 35, 570–580. 10.1521/suli.2005.35.5.570 16268773

[B19] Nock M. K. Hwang I. Sampson N. A. Kessler R. C. (2010). Mental disorders, comorbidity and suicidal behavior: results from the national comorbidity survey replication. Mol. Psychiatry 15, 868–876. 10.1038/mp.2009.29 19337207 PMC2889009

[B20] Olié E. Guillaume S. Jaussent I. Courtet P. Jollant F. (2010). Higher psychological pain during a major depressive episode may be a factor of vulnerability to suicidal ideation and act. J. Affect. Disord. 120, 226–230. 10.1016/j.jad.2009.03.013 19394086

[B21] Orbach I. Mikulincer M. Sirota P. Gilboa-Schechtman E. (2003). Mental pain: a multidimensional operationalization and definition. Suicide Life Threat. Behav. 33, 219–230. 10.1521/suli.33.3.219.23219 14582833

[B22] Patterson A. A. Holden R. R. (2012). Psychache and suicide ideation among men who are homeless: a test of Shneidman's model. Suicide Life Threat. Behav. 42, 147–156. 10.1111/j.1943-278X.2011.00078.x 22324750

[B23] Pereira E. J. Kroner D. G. Holden R. R. Flamenbaum R. (2010). Testing Shneidman's model of suicidality in incarcerated offenders and in undergraduates. Pers. Individ. Dif. 49, 912–917. 10.1016/j.paid.2010.07.029

[B24] Phillips M. R. Li X. Y. Zhang Y. P. (2002). Suicide rates in China, 1995-99. Lancet 359, 835–840. 10.1016/S0140-6736(02)07954-0 11897283

[B25] Pompili M. (2010). Exploring the phenomenology of suicide. Suicide Life Threat. Behav. 40, 234–244. 10.1521/suli.2010.40.3.234 20560745

[B26] Reisch T. Seifritz E. Esposito F. Wiest R. Valach L. Michel K. (2010). An fMRI study on mental pain and suicidal behavior. J. Affect. Disord. 126, 321–325. 10.1016/j.jad.2010.03.005 20434779

[B27] Scherer K. R. (2005). What are emotions? And how can they be measured?. Soc. Sci. Inform. 44, 695–729. 10.1177/0539018405058216

[B28] Shah A. Bhat R. McKenzie S. Koen C. (2009). Elderly suicide rates: cross-national comparisons and association with sex and elderly age-bands. Med. Sci. Law 47, 244–252. 10.1258/rsmmsl.47.3.244 17725239

[B29] Shneidman E. (2005). How I read. Suicide Life Threat. Behav. 35, 117–120. 10.1521/suli.35.2.117.62879 15843329

[B30] Shneidman E. S. (1998). Perspectives on suicidology: further reflections on suicide and psychache. Suicide Life Threat. Behav. 28, 245–250. 9807770

[B31] Shneidman E. S. (2001). Suicidology and the university: a founder's reflections at 80. Suicide Life Threat. Behav. 31, 1–8. 10.1521/suli.31.1.1.21314 11326763

[B32] Stewarta S. M. Felicea E. Claassena C. Kennarda B. D. Leeb P. W. Emslie G. J. (2006). Adolescent suicide attempters in Hong Kong and the United States. Soc. Sci. Med. 63, 296–306. 10.1016/j.socscimed.2006.01.005 16473448

[B33] Troister T. D'Agata M. T. Holden R. R. (2015). Suicide risk screening: comparing the beck depression inventory-II, beck hopelessness scale, and psychache scale in undergraduates. Psychol. Assess. 27, 1500–1506. 10.1037/pas0000126 25915787

[B34] Troister T. Holden R. R. (2010). Comparing psychache, depression, and hopelessness in their associations with suicidality: a test of Shneidman's theory of suicide. Pers. Individ. Differ. 49, 689–693. 10.1016/j.paid.2010.06.006

[B35] Troister T. Holden R. R. (2012). A two-year prospective study of psychache and its relationship to suicidality among high-risk undergraduates. J. Clin. Psychol. 68, 1019–1027. 10.1002/jclp.21869 22644790

[B36] van Heeringen K. Van den Abbeele D. Vervaet M. Soenen L. Audenaert K. (2010). The functional neuroanatomy of mental pain in depression. Psychiatry Res. 181, 141–144. 10.1016/j.pscychresns.2009.07.011 20074915

[B37] Xie W. Li H. Luo X. Fu R. Ying X. Wang N.. (2014). Anhedonia and pain avoidance in the suicidal mind: behavioral evidence for motivational manifestations of suicidal ideation in patients with major depressive disorder. J. Clin. Psychol. 70, 681–692. 10.1002/jclp.22055 24307489

[B38] Ye R. F. Geng Q. S. Chen J. Ou L. M. Zhang M. L. Dong C. L. (2013). Comparison of HADS and BDI for detecting depression in general hospital outpatients. Chin. J. Clin. Psychol. 21, 48–50.

[B39] Zhang J. Conwell Y. Zhou L. Jiang C. (2005). Culture, risk factors and suicide in rural china: a psychological autopsy case control study. Acta Psychiatr. Scand. 110, 430–437. 10.1111/j.1600-0447.2004.00388.x 15521827 PMC2730492

[B40] Zhang Y. Wang Y. Qian M. (1990). Reliability and validity of Beck Depression Inventory (BDI) examined in chinese samples. Chin. Ment. Health J. 4, 164–168.

